# **Postoperative pain relief after laparoscopic cholecystectomy: intraperitoneal sodium bicarbonate versus normal saline**

**Published:** 2016

**Authors:** Karim Saadati, Mohammad Reza Razavi, Daryoush Nazemi Salman, Shahrzad Izadi

**Affiliations:** 1 *Department of Thoracic Surgery, Ayatollah Mousavi Hospital, Zanjan University of Medical sciences, Zanjan, Iran.*; 2 *Nursing Care Research Center, Semnan University of Medical Sciences, Semnan, Iran.*; 3 *Department of Surgery, Ayatollah Mousavi Hospital, Zanjan University of Medical sciences, Zanjan, Iran.*; 4 *Department of Surgery, Kowsar Hospital, Semnan University of Medical sciences, Semnan, Iran *

**Keywords:** Sodium bicarbonate, Intraperitoneal irrigation, Laparoscopic cholecystectomy, Postoperative pain

## Abstract

**Aim:**

The aim of this study was to determine the effect of sodium bicarbonate irrigation versus normal saline irrigation in patients undergoing a laparoscopic cholecystectomy.

**Background:**

Pain in patients undergoing laparoscopic cholecystectomy is the most common complaint, especially in the abdomen, back, and shoulder region.

**Patients and methods:**

In a double blind randomized clinical trial, 150 patients were assigned to the three groups (50 patients in each group). Group A received intraperitoneal irrigation normal saline (NS). Groups B and C received irrigation sodium bicarbonate and none irrigation, respectively. Pain was assessed using a visual analog scale (VAS) for 6, 18 and 24 hours postoperatively, as well as one week after the surgery. Data analysis was performed using SPSS ver18 and chi-square, Fisher’s Exact Test, on-way ANOVA and repeated measure ANOVA tests.

**Results:**

Patients in groups showed no significant difference in terms of age, gender, past medical history and smoking history (p>0.05). Left shoulder tip pain was significantly lower only between the sodium bicarbonate group and non-washing group at 6, 18, and 24 hours postoperatively (P=0.04, P=0.02 and P=0.009 respectively). There was no significant difference between the three treatment groups in right shoulder tip pain, back pain and port site incisional pain.

**Conclusion:**

In laparoscopic cholecystectomy, peritoneal irrigation with sodium bicarbonate may reduce the intensity of postoperative shoulder tip pain and is an effective method for improving the quality of life within the early recovery period.

## Introduction

 Laparoscopic cholecystectomy is considered as the treatment of choice for symptomatic cholelithiasis (1). Laparoscopic surgery has displayed advantages over open surgery, including less post-operative pain, smaller incisions, shorter postoperative ileus, reduced blood loss, reduced length of hospital stay, faster recovery, as well as earlier return to preoperative activity and work (2-4). In fact, reduced postoperative pain is one of the biggest advantages of laparoscopy compared with open surgery. However, postoperative pain is not completely disappeared and is still considerable (5). Pain can increase morbidity and is the primary reason for prolonged hospitalization after laparoscopic cholecystectomy (6, 7). Patients frequently complain of back, shoulder region pains and discomfort of port site incisions(8). Shoulder and sub-diaphragmatic pain occurs in about 12% to 60% of patients (9). Peak of pain intensity is during the first few postoperative hours and usually declines after 2 or 3 days (10).

The etiology of pain after laparoscopic cholecystectomy is multifactorial (9). One suggested cause of pain after laparoscopy is the peritoneal insufflation with CO_2 _and phrenic nerve irritation in the peritoneal cavity (11-13). In fact, the acid milieu created by the dissolution of CO_2_ gas cause peritoneal irritation and phrenic nerve damage in laparoscopic cholecystectomy. Additional contributing factors include sociocultural status, and individual factors (9).

To date, administration of non-steroidal anti-inflammatory drugs (NSAIDs) and narcotics, gas drainages, intraperitoneal saline and intraperitoneal of local anesthetics and opioids were carried out to reduce pain after a laparoscopic cholecystectomy. While, use of these methods for pain relief after laparoscopic cholecystectomy had a lot of side effects or was not associated with a similar result (9, 14-17). Therefore, the clinical significance of pain control after laparoscopic surgery remains controversial.

This randomized clinical trial was designed to determine whether it is possible to reduce post laparoscopic pain by neutralizing acidic peritoneal environment (created by CO_2_ insufflation) using peritoneal wash out with sodium bicarbonate (an alkaline solution). 

## Patients and Methods

This study is a double blind randomized clinical trial (IRCT code: IRCT2015091123981N1r1), which was done from September 2012 to September 2014. After approval of the local ethics committee, 150 patients (80% power, 95% CI) of ASA physical status I-II undergoing elective laparoscopic cholecystectomy were prospectively randomized (with permutation block randomization) into 3 groups with concealment of the random group. Exclusion criteria were positive history of the use of opioids, steroids, NSAIDs and alcohol. Also, patients with acute cholecystitis, gangrene or empyema of the gallbladder, conversion to an open procedure for any reason, rupture of the gallbladder and bile leakage at the site of surgery, and bleeding in the site of surgery that could cause the peritoneal irritation were excluded. Demographic data, including age, gender, past medical history, smoking history and time of pneumoperitoneum was recorded.

All patients were referred to the operating room without premedication. A standard anesthetic was used during surgery. All laparoscopic cholecystectomy were performed by the same surgeon. A standard operative method was used with a 4-trocar technique in all patients. Nasogastric tube was inserted for all patients after intubation and was removed at the end of the surgery. Pneumoperitoneum was achieved with insufflations of CO_2 _through a periumbilical trocar, and was maintained at 14mm Hg during the entire surgical procedure. After removal of the gallbladder, hemostasis was performed in the surgical bed, and patients were randomly assigned to each of the three groups of 50 patients: 

Group A, normal saline group: 1000 ml normal saline 0.9%at a temperature of 37ْC was infused in the surgical bed, superior surface of the liver and under the right hemi diaphragm. 

Group B, sodium bicarbonate group: 50 ml sodium bicarbonate 7.5% in 1000 ml normal saline at a temperature of 37ْC was infused in the surgical bed, superior surface of the liver and under the right hemi diaphragm. 

Group C, non-washing group: no intraperitoneal irrigation was applied. 

In normal saline and bicarbonate groups, the fluid was completely aspirated before the pneumoperitoneum was deflated. CO_2_ was carefully evacuated at the end of the surgery by manual compression of the abdomen with open trocars (especially through the epigastrium trocar). No drains were used. Pain was assessed during an interview with the patients using a visual analog scale (VAS) of 0 to 10 by a trained interviewer unaware of the grouping of patients. During an interview, the interviewer explained the VAS for every patient, defining “0” for ‘no pain’ and “10” for ‘the worse imaginable pain’.

Assessments were done at the patient’s bedside at 6, 18 and 24 hours postoperatively, as well as a week after the surgery (in the first postoperative visit). Intensity of pain was recorded at 8 locations for assessment of port site incision pain, shoulder tip pain and back pain, including 3 port site incisions (port site incision pain), each of shoulder tips (shoulder tip pain) and the area between the shoulders and the tip of scapulas (Back pain).

During the first 24 hours after the surgery, all of the patients were allowed to receive Paracetamol as analgesic as needed (slow intra venous infusion of 1000 mg in 100 milliliter normal saline, not more than three times per 24 hours), and the exact times of administrations were recorded in the patients’ files in special tables for the study. 

Data were entered into a data bank using SPSS ver. 18. In addition to descriptive tables, in the analysis of the data we used chi-square, Fisher’s Exact Test, one-way ANOVA and repeated measure ANOVA. The statistical level of significance was set at P<0.05.

**Table 1:** Absolute and relative frequency distribution of the studied units based on demographic characteristics

characteristics	Sodium Bicarbonate Group (n=50)	Normal Saline Group (n=50)	Non Washing Group (n=50)	P Value	Test used
Age	44.98±12.6*	42.11±11.05	44.33±14.74	0.526	One-Way ANOVA
Gender					
Male Female	4(8)^†^ 46(92)	5 (10) 45(90)	3(6) 47(94)	0.929	Fisher's Exact Test
Past Medical History					
Ischemic Heart Disease Hypertension Diabetes Mellitus Dyspepsia Other Negative	1(2) 7(14) 1(2) 2(4) 9(18) 30(60)	0(0) 7(14) 3(6) 4(8) 5(10) 31(62)	1(2) 8(16) 4(8) 7(14) 4(8) 26(52)	0.460	Fisher's Exact Test
Smoking History					
Smoker Nonsmoker	3 (6) 47 (94)	2(4) 48(96)	1(2) 49(98)	0.871	Fisher's Exact Test
Time of pneumoperitoneum	50.04±11.22	56.21±15.22	45.18±10.88	0.000	One-Way ANOVA

* Mean ± standard deviation;

† Number (percent)

## Results

One hundred and fifty patients were enrolled into the study, 50 in each group. No significant difference was found between the 3 groups in terms of demographic data including: sex and age distribution, as well as past medical history (history of the ischemic heart disease, hypertension, diabetes mellitus, dyspepsia, etc.), and smoking history (Table 1). There was a significant difference in the average time of pneumoperitoneum between the non-washing and the normal saline groups.

**Table 2:** Pain intensity of all patients in the assessed sites

Time Points	Site of umbilical incision	Site of epigastrium incision	Site of subcostal incision	Left shoulder tip	Right shoulder tip	Area between the shoulders	Under the left scapula	Under the right scapula
At 6 hour	2.5(0-7)^*^	1.9(0-9)	1.7(0-6)	1.6(0-8)	1.69(0-8)	0.95(0-8)	0.11(0-6)	0.17(0-6)
At 18 hour	2.1(0-6)	1.6(0-8)	1.5(0-7)	1.5(0-8)	1.65(0-8)	0.73(0-6)	0.07(0-4)	0.17(0-6)
At 24 hour	1.7(0-5)	1.4(0-8)	1.3(0-8)	1.4(0-9)	1.6 (0-9)	0.55(0-6)	0.07(0-4)	0.15(0-6)
One week later	0.17(0-5)	0.17(0-4)	0.03(0-3)	0.05(0-5)	0.09(0-5)	0(0)	0(0)	0(0)

* Mean (range)

**Table 3:** Shoulder tip pain of the 3 groups at each time point

Group	Sodium bicarbonate group	Normal saline group	Non washing group
Left shoulder tip pain			
At 6 hour At 18 hour At 24 hour	1±1.9^*^ 0.9±1.7 0.8±1.7	1.5 (±2.4 1.48 (±2.1 1.5 (±2.2	2.2٭ (±2.7^†^ 2.1 ٭ (±2.4)^†^ 1.98 ٭ (±2.1)^†^
Right shoulder tip pain			
At 6 hour At 18 hour At 24 hour	1.5±2.1 1.5 (±2 1.52 (±2	1.5 (±2.3 1.7 (±2.1 1.82 (±2.3	2 (±2.6 2 (±2.4 1.8 (±1.9
Mean pain intensity of shoulders			
At 6 hour At 18 hour At 24 hour	1.3 (±1.9 1.2 (±1.7 1 (±1.7	1.52 (±2.2 1.6 (±1.9 1.7 (±2	2.1 (±2.6 1.94 (±2.3 2٭(±1.8^†^

* Mean ± standard deviation;

† Statistically significant difference vs. Sodium bicarbonate group (One-Way ANOVA)

**Table 4:** Number of analgesia demand over the 24 hours postoperatively

Group	Sodium bicarbonate group	Normal saline group	Non-washing group
Number of analgesia demand			
During the first 6 hour Between 6 and 18 hour Between 18 and 24 hour	0.8 (0-2) 0.62 (0-2) 0.25 (0-1)	0.84 (0-2) 0.7 (0-2) 0.3 (0-1)	0.86 (0-2) 0.74 (0-2) 0.33 (0-2)

Data are presented as mean (range)

There was no significant difference in the average time of pneumoperitoneum in sodium bicarbonate group and two other groups (Table 1). 

In our patients, the most painful site was the site of umbilical incision (the mean of pain intensity at 6, 18 and 24 hours postoperatively was 2.5, 2.1, and 1.7, respectively), followed by pain at other trocar sites and the shoulder tips. The area between the shoulders and under the scapulas had the lowest pain intensity (Table 2). Very few patients (six patients of non-washing group, four patients of normal saline group and three patients of bicarbonate group) had pain one week after the surgery that was in trocar sites and tip shoulders (Table 2).

***Port site incisional pain***

**Figure 1:**
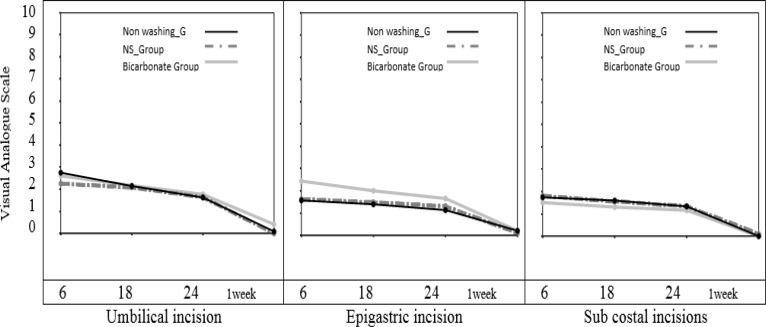
Changes of port site incisional pain over the time (repeated measure analysis of variance

In the evaluation of port site incisional pain, there was no statistically significant difference between the three treatment groups in ANOVA tests (figure 1).

***Shoulder tip pain***

**Figure 2:**
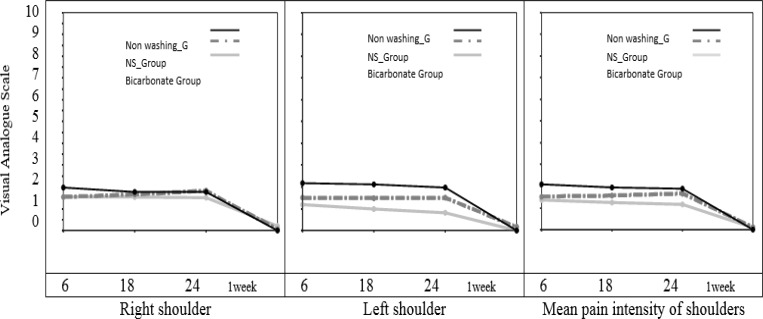
Changes of shoulder tip pain over the time (repeated measure analysis of variance

Comparison of shoulder tip pain with repeated measure analysis of variance showed that, overall pain intensity of left shoulder was significantly different between the three treatment groups. The sodium bicarbonate group had significantly less overall pain intensity than non-washing group (with "Between- subject effect test" P=0.02, F=3.625). Also change of pain intensity over the time was different between the 3 groups. Decrease of left shoulder tip pain over the time was more in sodium bicarbonate group in comparison with non-washing group (with "Within- subject effect test" P=0.023, F=2.469) (figure 2). Comparison of pain intensity of the three groups at each time point, by One-Way ANOVA revealed that; left shoulder tip pain was significantly lower in the sodium bicarbonate group in comparison with non-washing group, at 6, 18, and 24 hours postoperatively (with "Between- subject effect test", P=0.04, P=0.02 and P=0.009 respectively), and left shoulder tip pain was not significantly different between three groups one week after the surgery. Also, left shoulder tip pain was lower in the sodium bicarbonate group than in the normal saline group at 6, 18, and 24 hour after the surgery, but this pain did not reach statistical significance (table 3).

In the evaluation of the right shoulder tip pain, despite the fact that pain intensity of the sodium bicarbonate group was less than other two groups in almost all of the time points, there was no statistically significant difference in ANOVA tests (table 3).

Analysis using repeated measure ANOVA showed that there was no significant difference in the mean pain intensity of shoulders between the three groups (P = 0.17; figure 2). However, a steeper decreasing trend in the sodium bicarbonate group over time was observed. Also, evaluation of mean pain intensity of shoulders at each time point with One-Way ANOVA showed that pain intensity at 24 hour after the surgery in sodium bicarbonate group was significantly less than non-washing group (with "Between- subject effect test", P=0.04) (table 3).

***Back pain***

In the evaluation of the back pain (area between the shoulders and the tip of scapulas) there was no significant difference between the 3 treatment groups in ANOVA tests (figure 3).

***Postoperative analgesic requirement***

Regarding the number of analgesia demand over the 24 hours postoperatively, ANOVA tests showed no significant difference between the 3 treatment groups (table 4). 

## Discussion

Despite many advances in laparoscopic cholecystectomy, postoperative pain is still a serious problem, and in most reports, up to 80% of patients ask for analgesics after laparoscopic cholecystectomy (18). Pain intensity peaks over the first postoperative hours and gradually decreased over the following 48 to 72 hours (19). There are many evidences that the main source of pain after laparoscopic cholecystectomy is the peritoneum rather than the skin or abdominal wall (20). Due to high solubility and noncombustible properties, CO_2_ insufflation constitutes the commonest means of creating the pneumoperitoneum. CO_2 _pneumoperitoneum is known to cause systemic acidosis (21). Recent researches have shown that peritoneal acidosis could occur during CO_2_ laparoscopy (22-24). In one of the best designed studies, when CO_2_ gas was used for pneumoperitoneum, it was shown that the intraperitoneal pH was 6.0 immediately after the operation and raised to 6.4–6.7 and 6.8–6.9 on the first and second post-operation days, respectively (25). Peritoneal acidosis has been proposed as one of the most important factors responsible for damaging of the mesothelial lining of the peritoneum and consequent peritoneal irritation (23). Moreover, the phrenic nerve could be damaged by the acidic environment created by CO_2_ (13). Both of these factors lead to postoperative pain. In this study, we showed a reduction of postoperative pain in the intraperitoneal sodium bicarbonate wash out group. Sodium bicarbonate may neutralize effect of the acid milieu on peritoneal cavity and the phrenic nerve damage which consecutively will lead to a reduction of postoperative pain.

As you can see in figure 1 and 2, the most significant difference between the sodium bicarbonate group and the other treatment groups was seen in the shoulder tip pain. However, there was no significant intergroup difference in the trocar site pain. It is believed that, in laparoscopic cholecystectomy the trocar site pain is a somatic pain, whereas the shoulder tip pain is a visceral pain due to diaphragmatic irritation (26). Therefore, it seems that lower shoulder tip pain in the sodium bicarbonate group is due to reduction of peritoneal acidosis and a decrease in degree of irritation of peritoneum and phrenic nerve endings (15).

In previous studies that used sub diaphragmatic washout with saline solution, a significant decrease in postoperative pain intensity has been reported (12, 27). In those studies, the researchers concluded that one of the likely mechanisms for the positive effect of intraperitoneal saline washout on reduction of post laparoscopic pain has been the improved evacuation of residual carbon dioxide from the peritoneal cavity, especially sub diaphragmatic region (12, 27). In our study, a significant difference in the left shoulder tip pain and mean pain intensity of shoulders was observed between the sodium bicarbonate group and non-washing group. Also, lower left shoulder tip pain and mean pain intensity of shoulders and at 6, 18 and 24 hour after the surgery were found in the sodium bicarbonate group than in the normal saline group, but this did not reach statistical significance. We believe the mechanism that leads to lower shoulder tip pain in a sodium bicarbonate group in comparison with the two other groups is the following: the peritoneal irrigation with both of sodium bicarbonate and normal saline solutions reduces the CO_2_ gas above the liver, but sodium bicarbonate neutralizes the acidic sub diaphragmatic area more effective than saline solution that leads to less diaphragmatic and peritoneal irritation, as well as less postoperative pain.

In previous studies, which used intraperitoneal bupivacaine washout for reduction of pain after laparoscopic cholecystectomy, the effect was short-lived and did not exceed 6-12 hours after the surgery (28, 29). In a study by Morsy, et al., shoulder pain intensity measured by VAS after surgery was significantly lower in lidocaine and nalbuphine groups in comparison with the intraperitoneal normal saline group at first 8 h (8). In our study, the superiority of the sodium bicarbonate group over the non-washing group persisted within the first 24 hours after the surgery. Left shoulder tip pain was significantly lower in a sodium bicarbonate group at 6, 18 and 24 hours, postoperatively and mean pain intensity of shoulders in 24 hour after the surgery was also less in this group. However, in Morsey, et al. study, consumption of Paracetamol was significantly lower in lidocaine and nalbuphine groups. However, in our study there was no significant difference in the number of analgesia demand over the 24 hours postoperatively (8). 

The limitation of this study was the lack of integration groups in terms of age and sex. We recommend clinical trials studies with considering these factors due to effect of them on pain (30). 

Intraperitoneal irrigation of sodium bicarbonate is a simple and safe method that provides pain relief in the postoperative period after laparoscopic cholecystectomy compared with none-washing. Also, intraperitoneal irrigation of sodium bicarbonate was found to have a better pain relief profile compared with normal saline. We recommend that the sodium bicarbonate washout procedure to be used at laparoscopic cholecystectomy, because it is a way for reducing the post laparoscopic pain intensity for 24h after surgery.
